# Prevalence and correlates of depression, anxiety and trauma-like symptoms in Chinese psychiatric patients during the fifth wave of COVID-19 pandemic: a cross-sectional study in Hong Kong

**DOI:** 10.1186/s12888-024-05815-y

**Published:** 2024-05-17

**Authors:** Joe Kwun Nam Chan, Don Ho Hin Chang, Vivian Shi Cheng Fung, Eileena Mo Ching Chui, Corine Sau Man Wong, Ryan Sai Ting Chu, Yuen Kiu So, Jacob Man Tik Chan, Albert Kar Kin Chung, Krystal Chi Kei Lee, Calvin Pak Wing Cheng, Heidi Ka Ying Lo, Chi Wing Law, Wai Chi Chan, Wing Chung Chang

**Affiliations:** 1 Department of Psychiatry, School of Clinical Medicine, LKS Faculty of Medicine, The University of Hong Kong, Hong Kong, China; 2 Department of Psychiatry, Queen Mary Hospital, Hospital Authority, Hong Kong, China; 3 School of Public Health, LKS Faculty of Medicine, The University of Hong Kong, Hong Kong, China; 4 State Key Laboratory of Brain and Cognitive Sciences, The University of Hong Kong, Hong Kong, China; 5 Department of Psychiatry, The University of Hong Kong Queen Mary Hospital, Pokfulam, Hong Kong China

**Keywords:** Psychiatric patients, Mental disorders, COVID-19, Depression, Anxiety, Post-traumatic stress disorder

## Abstract

**Background:**

Psychiatric patients are susceptible to adverse mental health outcome during COVID-19 pandemic, but its associated factors are understudied. This observational cross-sectional study aimed to comprehensively examine prevalence and correlates of psychological distress, in terms of depression, anxiety and post-traumatic-stress-disorder (PTSD)-like symptoms, among Chinese adult psychiatric outpatients amidst the peak of fifth COVID-19 wave in Hong-Kong.

**Methods:**

A total of 415 patients (comprising 246 patients with common-mental-disorders [CMD] and 169 with severe-mental-disorders [SMD]) and 399 demographically-matched controls without mental disorders were assessed with self-rated questionnaires between 28-March and 8-April-2022, encompassing illness profile, mental health symptoms, psychosocial measures (loneliness, resilience, coping styles) and COVID-19 related factors. Univariate and multivariable logistic regression analyses were conducted to determine variables associated with moderate-to-severe depressive, anxiety and PTSD-like symptoms among psychiatric patients.

**Results:**

Our results showed that CMD patients had the greatest psychological distress relative to SMD patients and controls. Approximately 40–55% CMD patients and 25% SMD patients exhibited moderate-to-severe depression, anxiety and PTSD-like symptoms. Multivariable regression analyses revealed that female gender, lower educational attainment, single marital status, being housewife, more severe insomnia, psychotic-like symptoms and cognitive complaints, self-harm behavior, lower resilience, avoidance coping, never contracting COVID-19 infection, greater fear of contagion, and longer exposure to pandemic-related information were independently associated with depression, anxiety and/or PTSD-like symptoms in psychiatric patients.

**Conclusions:**

Our results affirm increased vulnerability of psychiatric patients toward psychological distress during pandemic. An array of identified correlates facilitates early detection of high-risk psychiatric patients for targeted strategies to minimize pandemic-related negative psychological impact.

**Supplementary Information:**

The online version contains supplementary material available at 10.1186/s12888-024-05815-y.

## Introduction

**Fig. 1:**
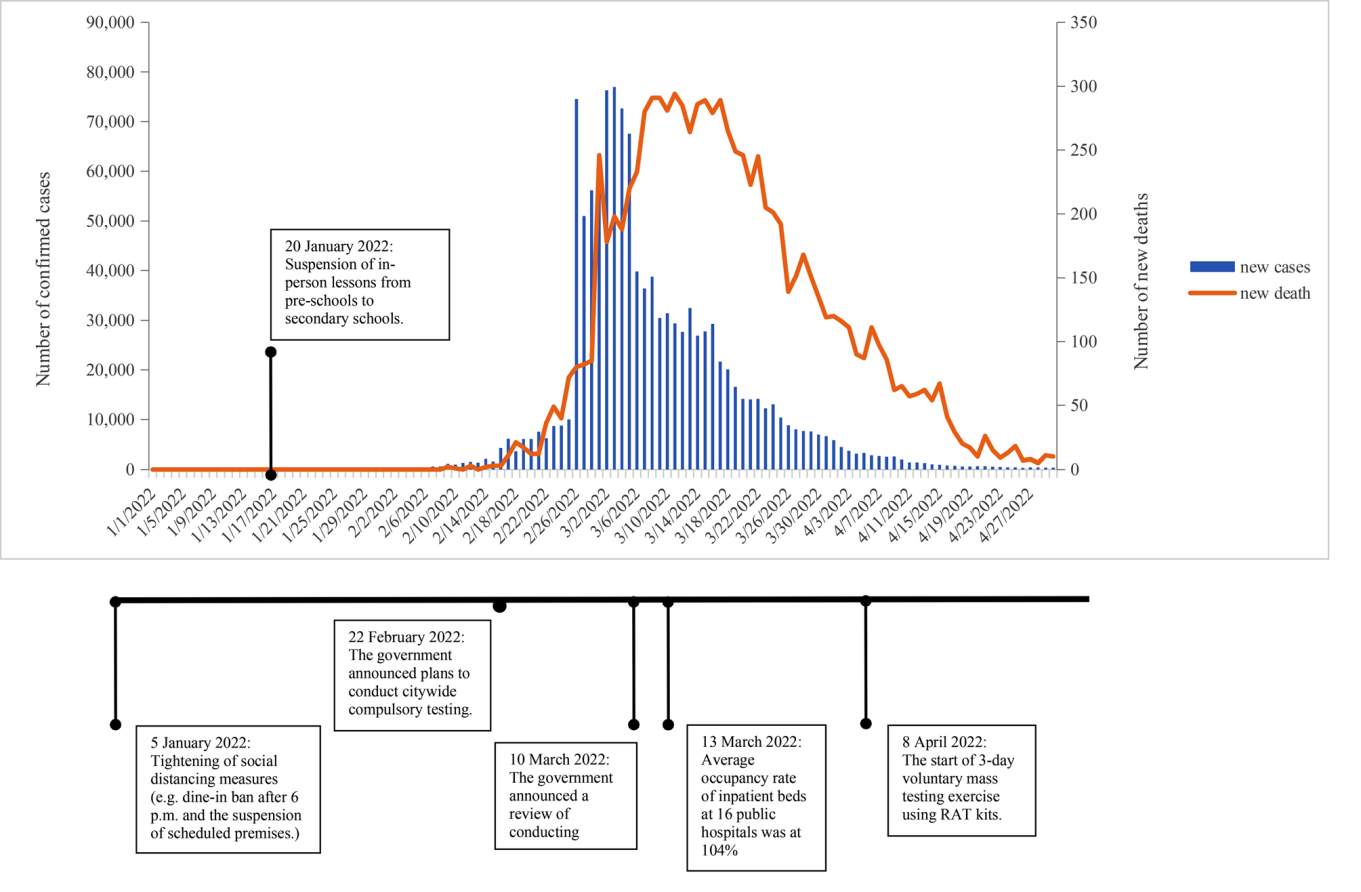
Chronology of the fifth wave of COVID-19 pandemic in Hong Kong and the number of daily confirmed cases and deaths

The COVID-19 pandemic caused by the SARS-CoV-2 virus has posed a significant threat to public health globally and locally. In Hong Kong (HK), the pandemic initially led to a fluctuating number of local infections until the emergence of the Omicron variant in late December 2021, which triggered the fifth wave of outbreaks. It was estimated that 60% of the population (4.4 million) was infected amid the fifth wave of COVID-19 pandemic [1] (Fig. 1). This wave led to a 7-day rolling average of COVID-19 related deaths reaching 3.73 per 1000 people at the peak, being the highest in the world [2, 3]. Owing to the highly contagious nature of the virus, various prevention measures have been implemented to contain its spread. However, accumulating research has consistently indicated that the pandemic and related physical-isolation strategies have resulted in adverse mental health outcomes, including depressive, anxiety and post-traumatic stress symptoms in the general population [4, 5].

Individuals with mental disorders are disproportionately affected by the pandemic and confinement policies due to disruption of critical factors in the illness management such as access to psychiatric care, and stability in daily routines, social rhythms and sleep patterns. Literatures has revealed that psychiatric patients had higher rates of contracting COVID-19 infection [6] and mortality [7, 8] than those without mental disorders. While investigations on the psychological impact of COVID-19 have primarily been conducted in the general population, limited research on psychiatric patients revealed that the presence of mental disorders was associated with higher levels of depression, anxiety and pandemic-related stress [9–14]. The prevalence of moderate-to-severe depression, anxiety and post-traumatic stress disorder (PTSD) symptoms in psychiatric patients during the pandemic could reach 40%, 25% and 30%, respectively [9, 11]. More than a quarter of psychiatric patients also reported re-emergence of mental health symptoms [15]. Of note, existing pandemic research on psychiatric patients was constrained by small sample size [11, 12, 14–17], self-report disorder without diagnostic reevaluation [9, 10, 13, 18], and treating psychiatric patients as a single broadly-defined category without further delineation into common and severe mental disorders [9, 11, 12, 15, 19]. Previous studies have revealed potential risk factors for adverse mental health outcomes in psychiatric patients during the COVID-19 pandemic including female gender, younger age, living alone, single marital status, unemployment/ income loss, increased substance/ alcohol use, loneliness, avoidant coping strategies, sleep disturbance, restricted access to psychiatric services, and fear of contracting infection, to name a few [9, 10, 18]. Other factors that were observed to be related to negative mental health outcomes in the general population during the pandemic included lower educational attainment, presence of chronic physical morbidity, and prolonged exposure to COVID-19 related information [20]. Nonetheless, due to a paucity of existing data regarding the correlates of psychological distress among psychiatric patients amidst the pandemic, further investigation is warranted to identify modifiable risk factors which would facilitate development of effective strategies to minimize such adverse mental health impact on this vulnerable population.

The current study aimed to examine the degrees of psychological distress, in terms of depressive, anxiety and PTSD-like symptom severity, in Chinese individuals with versus without pre-existing mental disorders during the peak of fifth COVID-19 wave in HK. Furthermore, we investigated the prevalence and correlates of moderate-to-severe depressive, anxiety and PTSD-like symptoms in individuals with common mental disorders (CMD) and severe mental disorders (SMD) separately. A wide array of variables encompassing socio-demographics, illness profile, mental health symptoms, psychosocial measures, and COVID-19 related factors were included in the analysis to ensure comprehensive evaluation.

## Methods

### Participants & settings

In this observational cross-sectional study, patients aged 18–64 years were recruited between March 28 and April 8, 2022 from adult psychiatric outpatient clinics in the Hong Kong West Cluster, a catchment area with a population of approximately 555,000. Patients who attended psychiatric follow-up were consecutively approached and recruited for survey assessment by research assistants after being screened by the designated nursing staff of the outpatient clinics via medical record checking to exclude those who were not eligible for the study (see exclusion criteria below). Patients were further categorized into those with common mental disorders (CMD, including depression and anxiety disorders) [21, 22] and those with severe mental disorders (SMD, including schizophrenia-spectrum disorders and other non-affective psychoses, and bipolar disorder) [7, 23]. To minimize the misclassification bias of psychiatric diagnosis (which was rated by patients in the survey), research psychiatrists had verified and ascertained patients’ principal diagnosis according to the International Classification of Diseases, 10th Revision (ICD-10) by reviewing medical records of psychiatric services (ICD-10 classification is used for psychiatric diagnostic assignment in HK public healthcare system). This medical review procedure also ensured that patients’ psychiatric diagnostic assignment preceded the fifth pandemic wave (i.e., status of pre-existing mental disorders). We then used data from another survey evaluating the psychological distress in the general population during the peak of the fifth pandemic wave in HK to derive a control group for comparison. We selected and matched controls from the general population survey to those in psychiatric patient sample using an exact matching method without replacement for age, gender and educational level on a one-to-one ratio. Individuals with any reported psychiatric diagnosis were excluded from control-group derivation. Exclusion criteria for all participants included learning disabilities, head injuries and neurological diseases (which may compromise cognitive function and comprehension ability for survey questions), as well as an inability to understand written Chinese language (as all survey questionnaires were Chinese version).The study was performed in accordance with the Declaration of Helsinki, and was approved by the Institutional Review Board of the University of Hong Kong/Hospital Authority Hong Kong West Cluster (HKU/HA HKW) and all participants provided written informed consent.

### Study assessment

The self-rated study assessment comprised five sections, including socio-demographics, illness profile, mental health symptoms, psychosocial measures, and COVID-19 related factors. Socio-demographics comprised age, gender, marital status, educational level, employment status, number of co-living individuals, size of residential area, and monthly household income. Illness profile included alcohol/ substance use disorder, and chronic physical disease.

Concerning mental health symptoms of both psychiatric patient and control groups, depressive and anxiety symptom severity were assessed by Patient Health Questionnaire-9 (PHQ-9) [24, 25] and Generalized Anxiety Disorder-7 scale (GAD-7) [26, 27], respectively, with both scales using a 4-point Likert scale ranging from 0 (never) to 3 (nearly every day). The total score for depression was ranging from 0 to 27, whereas that for anxiety was 0 to 21. A modified version of Impact of Event Scale-Revised (IES-R) [28, 29] specific to COVID-19 was administered to measure PTSD-like symptoms in a 5-point Likert scale (0 [never] to 4 [always]), with the total score ranging from 0 to 24. Insomnia symptoms were assessed using Insomnia Severity Index (ISI) [30, 31]. Positive symptoms and subjective cognitive impairment (SCI) were assessed only in psychiatric patients. Positive symptom subdomain items (4 items) of 15-item Community Assessment of Psychic Experiences Scale–Chinese version (CAPE-C15) [32] was employed to assess positive psychotic symptoms. Patients rated their frequency of positive symptoms on a 4-point Likert scale (1 [never] to 4 [nearly always]). We did not apply negative symptom subdomain items of CAPE-C15 to measure psychiatric patients’ negative symptoms as previous studies suggested considerable overlap with depressive symptoms [33, 34]. SCI was measured by a self-report questionnaire, adapted from Cognitive Complaints in Bipolar Disorder Rating Assessment (COBRA) [35, 36], which has been applied in a recent study examining SCI in psychiatric patients during COVID-19 lockdown [34]. The adapted SCI questionnaire comprised 5 items that reflected cognitive complaints manifested in everyday scenario including attention, processing speed, memory, learning and executive function (rated on frequency of cognitive complaints on a 4-point Likert scale, ranging from 0 [never] to 3 [nearly every day]). For all of the symptom scales, higher scores indicated greater symptom severity. Self-harm behavior during the fifth wave of COVID-19 was assessed.

Regarding psychosocial measures of both groups, loneliness was assessed by the UCLA 3-item Loneliness Scale [37] on a 3-point Likert scale (1 [hardly ever] to 3 [often]), with a higher score indicating greater loneliness. Resilience was assessed using the Brief Resilience Scale (BRS) [38] on a 5-point Likert scale (1 [strongly disagree] to 5 [strongly agree]), with higher scores indicating greater resilience. Participants’ coping strategies were assessed by an adapted Coping Orientation to Problems Experienced Inventory–Brief (Brief-COPE) [39], which used a 4-point Likert scale ranging from 0 (never) to 3 (always). The 14 items of the adapted Brief-COPE were grouped into 3 coping styles based on previous factor-analytic study [40], namely avoidant, emotion-focused and problem-focused coping styles for subsequent analysis. The SF-8 Health survey was used to assess health-related quality of life [41], on a 5-point Likert scale (1 [always] to 5 [never]), with a higher score indicating better quality of life. Evaluation of COVID-19 related factors comprised items assessing history of contracting COVID-19 infection, receipt of vaccination, fear of contagion, time spent on reading COVID-19 related information, COVID-19 related stressors experienced, specific infection control measures (under quarantine, mandatory COVID-19 testing) experienced and associated distress. Details of assessment of COVID-19 related factors are summarized in supplementary Table S1.

### Statistical analysis

We compared psychiatric patient and control groups (two-group comparison) as well as CMD, SMD and control groups (three-group comparison) in terms of levels of psychological distress, as indexed by three mental health outcomes comprising depressive, anxiety and PTSD-like symptoms. We also conducted two- and three-group comparisons on other variables for the sake of a complete overview of differential variations between controls and patients with CMD and SMD. Then, we categorize CMD patients and SMD patients into a subgroup with moderate-to-severe depressive, anxiety and PTSD-like symptoms on the basis of having a total score ≥ 10 on PHQ-9, GAD-7 and IES-R, respectively. To determine correlates of moderate-to-severe depression, anxiety and PTSD-like symptoms, we first performed three sets of a series of univariate binary logistic regression analyses, with each of the three mental health outcomes as dependent variable (depressed vs. non-depressed; anxiety vs. non-anxiety, PTSD-like symptoms vs. non-PTSD-like symptoms) and a wide range of socio-demographics, illness profile, other mental health symptoms (positive symptoms, insomnia symptoms, SCI), psychosocial measures (loneliness, resilience, coping styles, health-related quality of life) and COVID-19 related factors as independent variables. In addition to a priori potential confounders, i.e., age, gender and chronic physical diseases, those variables that were significantly related to the three mental health outcomes were included in the multivariable logistic regression models with backward stepwise approach to identify correlates independently associated with these mental health outcomes in CMD and SMD groups separately. Odds ratios (OR) with 95% confidence intervals (CI) were reported for all regression analyses. Multicollinearity of variables included in the multivariable regression models was evaluated using variance inflation factor (VIF), which was found to be within acceptable level (i.e., VIF < 10). As rate of missing data per selected study variables was low (ranged: 1-5%, with most < 3%), listwise deletion approach was used to handle missing data in multivariable regression analyses All statistical analyses were performed using R (4.1.2) version *glm2* package, and *P* < 0.05 was considered statistically significant.

## Results

### Sample characteristics

This study included a total of 415 psychiatric patients and 399 demographically-matched controls. Among the patient sample, 22.4%, 47.5% and 30.1% were aged 18–29 years, 30–49 years, and ≥ 50 years, respectively. Two hundred and sixty-two (63.1%) were female, and 171 (41.2%) had attained tertiary education or above. A total of 246 (59.3%) patients were diagnosed with CMD, while 169 (40.7%) were diagnosed with SMD. As presented in Table 1, psychiatric patients reported significantly more severe depression (PHQ-9 scores) and anxiety (GAD-7 scores) but milder PTSD-like symptoms (IES-R scores) than controls. CMD patients had more severe depression and anxiety than controls, and more severe depression, anxiety and PTSD-like symptoms than SMD patients. Controls had more severe PTSD-like symptoms than SMD patients. Otherwise, SMD patients and controls did not differ from each other in anxiety and depressive symptom severity. Table 1 summarizes other characteristics, including socio-demographics, illness profiles, other mental health symptoms, psychosocial measures and COVID-19 related factors between patients and controls, and among CMD patients, SMD patients and controls.

**Table 1:** Comparisons of characteristics between psychiatric patients and controls, and between CMD patients, SMD patients and controls

Characteristics	Controls (*n* = 399)	Psychiatric patients (*n* = 415)	*p* ^a^		CMD (*n* = 246)	SMD (*n* = 169)	*p* ^b^	Post-hoc comparison
Socio-demographics, n (%)								
Age, years			0.882				0.287	
18–29	90 (22.6)	93 (22.4)			61 (24.8)	32 (18.9)		
30–49	195 (48.9)	197 (47.5)			106 (43.1)	91 (53.8)		
≥ 50	114 (28.6)	125 (30.1)			79 (32.1)	46 (27.2)		
Female gender	258 (64.7)	262 (63.1)	0.969		163 (68.5)	99 (58.9)	0.140	
Marital status			< 0.001				< 0.001	
Single	178 (44.6)	220 (53.0)			105 (43.6)	115 (69.3)		SMD > CMD; SMD > Control
Married/ stable relationship	197 (49.4)	132 (31.8)			93 (38.6)	39 (23.5)		Control > CMD; CMD > SMD; Control > SMD
Divorced / widowed	24 (6.0)	55 (13.3)			43 (17.8)	12 (7.2)		CMD > Control; CMD > SMD
Educational attainment			0.865				0.667	
Secondary level or below	229 (57.4)	236 (56.9)			136 (56.2)	100 (60.6)		
Tertiary level or above	170 (42.6)	171 (41.2)			106 (43.8)	65 (39.4)		
Employment status			< 0.001				< 0.001	
Employed	241 (60.4)	208 (50.1)			127 (52.3)	81 (48.8)		Control > SMD
Full-time student	77 (19.3)	31 (7.5)			26 (10.7)	5 (3.0)		Control > CMD; Control > SMD; CMD > SMD
Unemployed	22 (5.5)	91 (21.9)			39 (16.0)	52 (31.3)		SMD > CMD; SMD > Control; CMD > Control
Housewife	38 (9.5)	61 (14.7)			47 (19.3)	14 (8.4)		CMD > Control; CMD > SMD
Retired	21 (5.3)	18 (4.3)			4 (1.6)	14 (8.4)		SMD > CMD
Living alone, yes	26 (6.5)	66 (15.9)	< 0.001		34 (13.8)	32 (18.9)	< 0.001	SMD > Control; CMD > Control
Housing area ^c^, sq. ft.			< 0.001				< 0.001	
≤ 500	211 (52.9)	297 (71.6)			185 (75.2)	112 (66.3)		SMD > Control; CMD > Control
> 500	188 (47.1)	105 (25.3)			54 (22.0)	51 (30.2)		Control > CMD; Control > SMD
Monthly household income ^d^, HKD ^e^			< 0.001				< 0.001	
≤ 25,000	145 (36.3)	268 (64.6)			149 (60.6)	119 (70.4)		CMD > Control; SMD > Control
> 25,000	254 (63.7)	128 (30.8)			87 (35.4)	41 (24.3)		Control > SMD; Control > CMD
Illness profiles, n (%)								
Alcohol / substance use disorders, yes	12 (3.0)	28 (6.7)	0.013		19 (7.7)	9 (5.3)	0.024	CMD > Control
Chronic physical disease, yes	73 (18.3)	118 (28.4)	< 0.001		78 (31.7)	40 (23.7)	< 0.001	CMD > Control
Mental health symptoms, mean (SD)								
Depressive symptoms	6.7 (6.0)	9.5 (7.4)	< 0.001		11.4 (7.2)	6.7 (6.7)	< 0.001	CMD > Control; CMD > SMD
Anxiety symptoms	5.8 (5.3)	7.6 (6.6)	< 0.001		9.2 (6.4)	5.3 (6.2)	< 0.001	CMD > Control; CMD > SMD
PTSD-like symptoms	8.4 (5.2)	7.3 (6.1)	< 0.001		8.5 (6.1)	5.5 (5.6)	< 0.001	Control > SMD; CMD > SMD
Insomnia symptoms	8.2 (5.4)	11.7 (7.4)	< 0.001		13.6 (7.2)	8.9 (6.8)	< 0.001	CMD > Control; CMD > SMD
Positive psychotic symptoms	-	5.6 (2.2)	-		5.6 (2.2)	5.6 (2.3)	0.969	
Subjective cognitive impairment	-	4.8 (4.2)	-		5.3 (4.1)	4.0 (4.1)	0.002	
Self-harm behaviour, n (%)	4 (1.0)	37 (9.0)	< 0.001		29 (12.0)	8 (4.8)	< 0.001	SMD > Control; CMD > Control; CMD > SMD
Psychosocial measures, mean (SD)								
Loneliness	4.4 (1.6)	4.9 (1.9)	< 0.001		5.1 (2.0)	4.7 (1.8)	< 0.001	CMD > SMD; CMD > Control
Resilience	19.8 (4.2)	17.2 (4.6)	< 0.001		16.5 (4.6)	18.3 (4.4)	< 0.001	Control > CMD; Control > SMD
Avoidant coping	9.0 (2.2)	4.9 (2.7)	< 0.001		5.5 (2.6)	4.0 (2.6)	< 0.001	Control > CMD; Control > SMD; CMD > SMD
Emotion-focused coping	12.2 (3.1)	6.8 (3.3)	< 0.001		7.2 (3.1)	6.3 (3.6)	< 0.001	Control > CMD; Control > SMD; CMD > SMD
Problem-focused coping,	6.6 (2.0)	3.8 (2.1)	< 0.001		4.0 (1.9)	3.6 (2.2)	< 0.001	Control > CMD; Control > SMD; CMD > SMD
Health-related quality of life	-	25.4 (6.2)	-		24.2 (5.9)	27.1 (6.2)	< 0.001	
COVID-19 related factors								
History of contracting COVID infection, yes	85 (21.3)	109 (26.3)	0.082		69 (28.0)	40 (23.7)	0.141	
Fear of COVID contagion, mean (SD)	4.7 (3.0)	4.5 (3.2)	0.420		4.7 (3.2)	4.3 (3.1)	0.357	
COVID-19 vaccine doses received			0.006				0.017	
0–1 dose	72 (18.0)	50 (12.0)			32 (13.0)	18 (10.7)		Control > SMD
2–3 doses	314 (78.7)	347 (83.6)			201 (81.7)	146 (86.4)		SMD > Control
Time spent on reading COVID-19 information			< 0.001				< 0.001	
None	174 (43.6)	255 (61.4)			150 (61.0)	105 (62.1)		CMD > Control; SMD > Control
1–3 h per day	168 (42.1)	94 (22.7)			52 (21.1)	42 (24.9)		Control > CMD; Control > SMD
≥ 4 h per day	56 (14.0)	55 (13.3)			38 (15.4)	17 (10.1)		
Number of COVID-19 related stressors ^f^			0.006				< 0.001	
0–2	317 (79.4)	294 (70.8)			161 (65.4)	133 (78.7)		SMD > CMD; Control > CMD
3–5	59 (14.8)	72 (17.3)			51 (20.7)	21 (12.4)		
6–8	21 (5.3)	44 (10.6)			31 (12.6)	13 (7.7)		CMD > Control
Mandatory testing or quarantine, yes	41 (10.3)	72 (17.3)	0.001		46 (18.7)	26 (15.4)	0.004	CMD > Control
Distress by social-distancing measures, mean (SD)	4.7 (3.4)	5.0 (3.3)	0.063		5.6 (3.3)	4.2 (3.2)	< 0.001	CMD > Control; CMD > SMD

*Note* CMD = common mental disorders; COVID = coronavirus; PTSD = post-traumatic stress disorders; SD = standard deviation; SMD = severe mental disorders

^a^ Two-group comparison (psychiatric patients and controls) was computed by independent samples t-tests and chi-square tests for continuous and categorical variables, respectively

^b^ Three-group comparison (CMD, SMD and control groups) was computed by one-way ANOVA (followed by Bonferroni post-hoc comparison if ANOVA result was statistically significant) and chi-square tests for continuous and categorical variables, respectively

^c^ The median of housing area excluding common area in Hong Kong is approximately 430 square feet according to the Population Census 2021

^d^ The median of monthly household income in Hong Kong is HKD27,640 according to the Population Census 2021

^e^ As of 21 Mar, 2023, 1 HKD = 0.13 USD

^f^ COVID-19 related stressors included finance, work, physical health, mental health, food and supplies, medical care and medication, family relationship and interpersonal relationships (see Table S1)

### Correlates of depression, anxiety and PTSD-like symptoms in CMD patients

Among CMD patients, prevalence of moderate-to-severe depressive, anxiety and PTSD-like symptoms was 55.7% (49.8–62.3%), 43.5% (37.7–50.2%) and 40.2% (34.6–46.9%), respectively. Univariate regression analyses revealed that employment status, insomnia and positive symptoms, SCI, self-harm behavior, loneliness, resilience, avoidant coping, fear of contagion, receipt of vaccination, COVID-19 related stressors and distress due to social-distancing measures were significantly related to all of the three mental health outcomes in CMD patients (Table 2). Living alone and emotion-focused coping were linked to depression and anxiety, whereas history of contracting COVID-19 was associated only with depression. Monthly household income was related to both depression and PTSD-like symptoms, while divorced /widowed status and exposure to COVID-19 related information were significantly associated with PTSD-like symptoms (Table 2).

**Table 2:** Univariate logistic regression analyses for depression, anxiety, and PTSD-like symptoms in patients with common mental disorders

Characteristics	Depression (*n* = 137)		Anxiety (*n* = 107)		PTSD-like symptoms (*n* = 99)	
	OR (95% CI)	*p*	OR (95% CI)	*p*	OR (95% CI)	*p*
Socio-demographics						
Age, years						
18–29	1 [reference]	-	1 [reference]	-	1 [reference]	-
30–49	1.19 (0.61–2.29)	0.611	0.69 (0.36–1.32)	0.265	1.82 (0.93–3.54)	0.079
≥ 50	0.70 (0.35–1.40)	0.319	0.53 (0.26–1.06)	0.074	1.61 (0.79–3.27)	0.192
Gender						
Male	1 [reference]	-	1 [reference]	-	1 [reference]	-
Female	1.50 (0.85–2.65)	0.166	1.71 (0.96–3.04)	0.068	1.50 (0.84–2.65)	0.167
Education attainment						
Secondary level or below	1 [reference]	-	1 [reference]	-	1 [reference]	-
Tertiary level or above	0.92 (0.54–1.55)	0.741	1.03 (0.61–1.73)	0.919	0.78 (0.47–1.32)	0.358
Marital status						
Single	1 [reference]	-	1 [reference]	-	1 [reference]	-
Married / stable relationship	0.93 (0.52–1.65)	0.794	0.89 (0.50–1.59)	0.700	1.04 (0.58–1.87)	0.884
Divorced / widowed	2.18 (0.99–4.81)	0.055	1.56 (0.75–3.25)	0.233	2.19 (1.06–4.54)	0.035
Employment status						
Employed	1 [reference]	-	1 [reference]	-	1 [reference]	-
Full-time student	2.00 (0.82–4.87)	0.129	1.90 (0.80–4.52)	0.148	1.15 (0.47–2.82)	0.753
Unemployed	12.35 (3.59–42.45)	< 0.001	3.79 (1.74–8.25)	< 0.001	5.35 (2.41–11.87)	< 0.001
Housewife	1.81 (0.91–3.60)	0.091	1.53 (0.77–3.06)	0.228	1.91 (0.95–3.83)	0.070
Retired	1.12 (0.15–8.23)	0.909	1.75 (0.24–12.86)	0.582	2.18 (0.30–16.05)	0.444
Living alone						
No	1 [reference]	-	1 [reference]	-	1 [reference]	-
Yes	0.42 (0.18–0.99)	0.047	0.38 (0.17–0.83)	0.015	0.71 (0.34–1.49)	0.371
Housing area, sq. ft.						
≤ 500	1 [reference]	-	1 [reference]	-	1 [reference]	-
> 500	1.05 (0.56–1.96)	0.888	0.90 (0.48–1.67)	0.736	0.73 (0.39–1.38)	0.332
Monthly household income, HKD						
≤ 25,000	1 [reference]	-	1 [reference]	-	1 [reference]	-
> 25,000	0.44 (0.25–0.75)	0.003	0.61 (0.35–1.05)	0.075	0.48 (0.28–0.85)	0.012
Illness profiles						
Alcohol / substance use disorders						
No	1 [reference]	-	1 [reference]	-	1 [reference]	-
Yes	1.77 (0.60–5.19)	0.301	1.73 (0.67–4.48)	0.256	2.09 (0.81–5.40)	0.129
Chronic physical disease						
No	1 [reference]	-	1 [reference]	-	1 [reference]	-
Yes	0.67 (0.38–1.16)	0.152	0.68 (0.39–1.19)	0.175	0.92 (0.52–1.60)	0.757
Mental health symptoms						
Insomnia symptoms	1.28 (1.20–1.37)	< 0.001	1.22 (1.16–1.29)	< 0.001	1.17 (1.12–1.23)	< 0.001
Positive psychotic symptoms	1.96 (1.53–2.51)	< 0.001	1.90 (1.54–2.33)	< 0.001	1.59 (1.34–1.88)	< 0.001
Subjective cognitive impairment	1.61 (1.43–1.82)	< 0.001	1.43 (1.30–1.57)	< 0.001	1.33 (1.22–1.44)	< 0.001
Self-harm						
No	1 [reference]	-	1 [reference]	-	1 [reference]	-
Yes	7.12 (2.08–24.33)	0.002	5.70 (2.23–14.62)	< 0.001	2.71 (1.22–6.03)	0.015
Psychosocial measures						
Loneliness	1.79 (1.50–2.14)	< 0.001	1.77 (1.50–2.09)	< 0.001	1.45 (1.26–1.67)	< 0.001
Resilience	0.74 (0.68–0.80)	< 0.001	0.75 (0.69–0.81)	< 0.001	0.81 (0.76–0.86)	< 0.001
Avoidant coping	1.42 (1.25–1.62)	< 0.001	1.55 (1.34–1.79)	< 0.001	1.31 (1.16–1.47)	< 0.001
Emotion-focused coping	1.11 (1.01–1.21)	0.023	1.14 (1.04–1.24)	0.005	1.05 (0.96–1.14)	0.287
Problem-focused coping	0.98 (0.86–1.12)	0.765	1.04 (0.91–1.19)	0.556	0.95 (0.83–1.09)	0.477
Heath-related quality of life	1.00 (1.00–1.00)	0.456	1.00 (1.00–1.00)	0.917	1.00 (1.00–1.00)	0.590
COVID-19 related factors						
History of contracting COVID infection						
No	1 [reference]	-	1 [reference]	-	1 [reference]	-
Yes	0.45 (0.25–0.80)	0.007	0.63 (0.35–1.13)	0.120	0.60 (0.33–1.08)	0.088
Fear of COVID contagion	1.16 (1.07–1.27)	< 0.001	1.15 (1.05–1.25)	0.002	1.28 (1.16–1.41)	< 0.001
COVID-19 vaccine doses received						
0–1 dose	1 [reference]	-	1 [reference]	-	1 [reference]	-
2–3 doses	0.39 (0.16–0.96)	0.040	0.39 (0.18–0.85)	0.018	0.44 (0.20–0.94)	0.034
Time spent on reading COVID-19 information						
None	1 [reference]	-	1 [reference]	-	1 [reference]	-
1–3 h per day	1.07 (0.55–2.05)	0.847	0.91 (0.47–1.75)	0.771	2.13 (1.12–4.05)	0.021
≥ 4 h per day	1.82 (0.83–3.97)	0.134	2.14 (1.01–4.51)	0.046	1.83 (0.88–3.81)	0.108
Number of COVID-19 related stressors						
0–2	1 [reference]	-	1 [reference]	-	1 [reference]	-
3–5	16.24 (5.56–47.45)	< 0.001	6.25 (3.07–12.73)	< 0.001	3.38 (1.74–6.55)	< 0.001
6–8	39.86 (5.28–300.87)	< 0.001	12.50 (4.50–34.73)	< 0.001	12.67 (4.57–35.14)	< 0.001
Mandatory testing or quarantine						
No	1 [reference]	-	1 [reference]	-	1 [reference]	-
Yes	0.64 (0.32–1.25)	0.190	1.03 (0.53–1.99)	0.938	0.82 (0.42–1.60)	0.565
Distress by social-distancing measures	1.30 (1.18–1.43)	< 0.001	1.26 (1.15–1.39)	< 0.001	1.20 (1.10–1.31)	< 0.001

*Note* CI = confidence interval; COVID = coronavirus; HKD = Hong Kong dollars; OR = odds ratio

Table 3 summarizes the results of the final multivariate regression models on three mental health outcomes. More severe insomnia symptoms and SCI, lower resilience, never contracting COVID-19 infection, and greater number of COVID-19 related stressors were significantly associated with depression. Younger age, more severe insomnia symptoms, higher levels of positive symptoms, SCI and lower resilience were significantly associated with anxiety. More severe insomnia symptoms, higher levels of SCI and fear of COVID-19 contagion were significantly related to PTSD-like symptoms.

**Table 3:** Multivariable logistic regression for depression, anxiety, and PTSD-like symptoms in patients with common mental disorders a

Characteristics	OR (95% CI)	*p*
Final model for depression		
Insomnia symptoms	1.19 (1.08–1.31)	0.001
Subjective cognitive impairment	1.30 (1.10–1.54)	0.002
Resilience	0.85 (0.76–0.96)	0.008
History of contracting COVID-19 infection		
No	1 [reference]	-
Yes	0.34 (0.13–0.93)	0.036
Number of COVID-19 related stressors		
0–2	1 [reference]	-
3–5	6.87 (1.74–27.14)	0.006
6–8	8.09 (0.77–85.20)	0.082
Final model for anxiety		
Age, years		
18–29	1 [reference]	-
30–49	0.39 (0.15–1.05)	0.062
≥ 50	0.13 (0.04–0.50)	0.003
Insomnia symptoms	1.11 (1.03–1.20)	0.010
Positive psychotic symptoms	1.39 (1.06–1.83)	0.018
Subjective cognitive impairment	1.22 (1.06–1.40)	0.005
Resilience	0.83 (0.74–0.93)	0.001
Avoidant coping	1.25 (1.01–1.54)	0.041
Final model for PTSD-like symptoms		
Insomnia	1.09 (1.02–1.16)	0.016
Subjective cognitive impairment	1.21 (1.07–1.36)	0.002
Resilience	0.91 (0.82–1.00)	0.051
Fear of COVID-19 contagion	1.19 (1.05–1.36)	0.007

*Abbreviations* CI = confidence interval; COVID = coronavirus; OR = odds ratio

^a^ Only those variables that were retained in the final regression models are presented

### Correlates of depression, anxiety and PTSD-like symptoms in SMD patients

For SMD patients, prevalence of moderate-to-severe depression, anxiety and PTSD-like symptoms was 24.3% (18.6–31.7%), 23.1% (17.5–30.4%) and 24.3% (18.6–31.7%), respectively. Univariable regression analyses showed that marital status, SCI, insomnia and positive symptoms, self-harm behavior, loneliness, resilience, avoidant coping, fear of contagion, COVID-19 related stressors and distress due to social-distancing measures were significantly related to all of these three mental health outcomes (Table 4). Educational attainment and substance /alcohol use disorder were related to both depression and anxiety, while exposure to COVID-19 related information was related to anxiety and PTSD-like symptoms. Furthermore, depression was linked to housing area, whereas anxiety was associated with monthly household income, emotion-focused coping and health-related quality of life. Other variables that were also related to PTSD-like symptoms included gender and employment status (Table 4).

**Table 4:** Univariate binary logistic regression analyses for depression, anxiety, and PTSD-like symptoms in patients with severe mental disorders

Characteristics	Depression (*n* = 41)		Anxiety (*n* = 39)		PTSD-like symptoms (*n* = 41)	
	OR (95% CI)	*p*	OR (95% CI)	*p*	OR (95% CI)	*p*
Socio-demographics						
Age, years						
18–29	1 [reference]	-	1 [reference]	-	1 [reference]	-
30–49	0.96 (0.38–2.45)	0.929	0.97 (0.38–2.47)	0.950	2.55 (0.81–8.02)	0.110
≥ 50	1.02 (0.35–2.94)	0.971	0.82 (0.28–2.43)	0.718	2.84 (0.83–9.73)	0.096
Gender						
Male	1 [reference]	-	1 [reference]	-	1 [reference]	-
Female	1.09 (0.53–2.26)	0.809	1.54 (0.73–3.28)	0.260	3.08 (1.36–6.98)	0.007
Educational attainment						
Secondary level or below	1 [reference]	-	1 [reference]	-	1 [reference]	-
Tertiary level or above	0.29 (0.12–0.67)	0.004	0.21 (0.08–0.53)	0.001	0.57 (0.26–1.22)	0.145
Marital status						
Single	1 [reference]	-	1 [reference]	-	1 [reference]	-
Married/stable relationship	1.62 (0.70–3.75)	0.262	2.99 (1.31–6.83)	0.009	2.73 (1.23–6.07)	0.014
Divorced / widowed	5.74 (1.49–22.05)	0.011	3.92 (1.09–14.10)	0.037	1.33 (0.33–5.32)	0.684
Employment status						
Employed	1 [reference]	-	1 [reference]	-	1 [reference]	-
Full-time student	0.93 (0.10–8.84)	0.947	0.86 (0.09–8.20)	0.897	N/A	N/A
Unemployed	2.15 (0.98–4.74)	0.057	1.30 (0.58–2.93)	0.521	1.29 (0.56–2.94)	0.548
Housewife	1.24 (0.30–5.07)	0.769	1.53 (0.42–5.56)	0.517	5.02 (1.53–16.43)	0.008
Retired	-	-	0.31 (0.04–2.60)	0.282	0.68 (0.14–3.39)	0.642
Living alone						
No	1 [reference]	-	1 [reference]	-	1 [reference]	-
Yes	1.19 (0.47–3.04)	0.713	1.06 (0.42–2.71)	0.902	0.91 (0.37–2.24)	0.836
Housing area, sq. ft.						
≤ 500	1 [reference]	-	1 [reference]	-	1 [reference]	-
> 500	0.33 (0.13–0.80)	0.015	0.57 (0.25–1.32)	0.188	0.90 (0.42–1.96)	0.793
Monthly household income, HKD						
≤ 25,000	1 [reference]	-	1 [reference]	-	1 [reference]	-
> 25,000	0.53 (0.21–1.32)	0.173	0.36 (0.13–0.99)	0.047	0.64 (0.27–1.54)	0.323
Illness profiles						
Alcohol / substance use disorder						
No	1 [reference]	-	1 [reference]	-	1 [reference]	-
Yes	6.63 (1.58–27.88)	0.010	4.41 (1.12–17.34)	0.034	1.02 (0.20–5.25)	0.984
Chronic physical disease						
No	1 [reference]	-	1 [reference]	-	1 [reference]	-
Yes	1.26 (0.56–2.86)	0.573	1.00 (0.42–2.34)	0.991	2.07 (0.94–4.53)	0.070
Mental health symptoms						
Insomnia symptoms	1.26 (1.16–1.36)	< 0.001	1.24 (1.15–1.34)	< 0.001	1.14 (1.08–1.21)	< 0.001
Positive psychotic symptoms	1.79 (1.46–2.20)	< 0.001	1.69 (1.40–2.05)	< 0.001	1.37 (1.17–1.61)	< 0.001
Subjective cognitive impairment	1.56 (1.36–1.79)	< 0.001	1.45 (1.29–1.64)	< 0.001	1.40 (1.26–1.57)	< 0.001
Self-harm						
No	1 [reference]	-	1 [reference]	-	1 [reference]	-
Yes	10.11 (1.95–52.36)	0.006	11.18 (2.16–57.98)	0.004	1.96 (0.45–8.60)	0.371
Psychosocial measures						
Loneliness	1.86 (1.47–2.35)	< 0.001	1.78 (1.42–2.23)	< 0.001	1.59 (1.29–1.96)	< 0.001
Resilience	0.80 (0.73–0.86)	< 0.001	0.80 (0.74–0.87)	< 0.001	0.86 (0.81–0.92)	< 0.001
Avoidant coping	1.33 (1.14–1.55)	< 0.001	1.41 (1.20–1.66)	< 0.001	1.24 (1.07–1.43)	0.004
Emotion-focused coping	1.09 (0.98–1.21)	0.122	1.16 (1.04–1.30)	0.009	1.09 (0.98–1.21)	0.115
Problem-focused coping	1.00 (0.85–1.18)	0.995	1.12 (0.95–1.32)	0.178	1.16 (0.99–1.37)	0.067
Health-related quality of life	1.00 (1.00–1.00)	0.774	0.80 (0.73–0.87)	< 0.001	1.00 (1.00–1.00)	0.674
COVID-19 related factors						
History of contracting COVID-19 infection						
No	1 [reference]	-	1 [reference]	-	1 [reference]	-
Yes	1.53 (0.69–3.42)	0.299	1.17 (0.51–2.69)	0.712	0.95 (0.41–2.22)	0.906
Fear of COVID-19 contagion	1.14 (1.02–1.29)	0.028	1.27 (1.11–1.44)	< 0.001	1.35 (1.18–1.55)	< 0.001
COVID-19 vaccines doses received						
0–1 dose	1 [reference]	-	1 [reference]	-	1 [reference]	-
2–3 doses	0.59 (0.20–1.71)	0.329	0.54 (0.19–1.55)	0.248	0.43 (0.15–1.19)	0.105
Time spent on reading COVID-19 information						
None	1 [reference]	-	1 [reference]	-	1 [reference]	-
1–3 h per day	1.89 (0.84–4.21)	0.122	2.39 (1.05–5.44)	0.038	3.06 (1.34–6.98)	0.008
≥ 4 h per day	1.46 (0.46–4.59)	0.519	2.52 (0.82–7.69)	0.106	7.86 (2.61–23.68)	< 0.001
Number of COVID-19 related stressors						
0–2	1 [reference]	-	1 [reference]	-	1 [reference]	-
3–5	32.51 (8.68–121.76)	< 0.001	17.66 (5.99–52.09)	< 0.001	7.86 (2.92–21.19)	< 0.001
6–8	4.78 (1.33–17.25)	0.017	14.13 (3.81–52.32)	< 0.001	19.65 (4.95–78.01)	< 0.001
Mandatory testing or quarantine						
No	1 [reference]	-	1 [reference]	-	1 [reference]	-
Yes	0.96 (0.35–2.62)	0.936	1.20 (0.46–3.14)	0.705	1.03 (0.38–2.80)	0.956
Distress by social-distancing measures	1.18 (1.05–1.33)	0.004	1.16 (1.03–1.30)	0.014	1.20 (1.07–1.35)	0.002

*Note* CI = confidence intervals; COVID = coronavirus; N/A = not available due to small cell size; OR = odds ratio

As shown in Table 5, educational attainment (secondary level or below), single marital status, more severe SCI and self-harm behavior were independently associated with depression. Anxiety was significantly associated with attaining secondary education level or below, more severe insomnia symptoms, higher levels of engagement in avoidant coping, and longer exposure to COVID-19 related information. Lastly, female gender, employment status (being housewife), higher levels of SCI and fear of contagion were significantly associated with PTSD-like symptoms.

**Table 5:** Multivariable logistic regression for depression, anxiety, and PTSD-like symptoms in patients with severe mental disorders a

Characteristics	OR (95% CI)	*p*
Final model for depression		
Educational attainment		
Secondary level or below	1 [reference]	-
Tertiary level or above	0.06 (0.01–0.45)	0.006
Marital status		
Single	1 [reference]	-
Married/stable relationship	0.15 (0.02–0.94)	0.043
Divorced / widowed	4.88 (0.27–87.41)	0.282
Positive psychotic symptoms	1.38 (1.02–1.87)	0.040
Subjective cognitive impairment	1.63 (1.27–2.09)	< 0.001
Resilience	0.75 (0.59–0.94)	0.014
Final model for anxiety		
Educational attainment		
Secondary level or below	1 [reference]	-
Tertiary level or above	0.01 (0.01–0.15)	< 0.001
Insomnia symptoms	1.29 (1.10–1.51)	0.002
Avoidant coping	1.55 (1.09–2.19)	0.014
Health-related quality of life	0.77 (0.65–0.90)	0.001
Time spent on reading COVID-19 information		
None	1 [reference]	-
1–3 h per day	7.80 (1.26–48.51)	0.028
≥ 4 h per day	2.60 (0.24–28.58)	0.436
Final model for PTSD-like symptoms		
Gender		
Male	1 [reference]	-
Female	9.54 (1.64–55.38)	0.012
Employment status		
Employed	1 [reference]	-
Full-time student	N/A	N/A
Unemployed	1.61 (0.34–7.57)	0.547
Housewife	28.68 (3.01–273.44)	0.004
Retired	4.02 (0.50–32.40)	0.192
Subjective cognitive impairment	1.57 (1.25–1.97)	< 0.001
Number of COVID-19 related stressors		
0–2	1 [reference]	-
3–5	0.72 (0.12–4.45)	0.727
6–8	15.10 (1.70–134.46)	0.015

*Abbreviations* CI = confidence intervals; COVID = coronavirus; N/A = not available due to small cell size; OR = odds ratio

^a^ Only those variables that were retained in final regression models are presented

## Discussion

To our knowledge, this is among the few studies comprehensively evaluating a wide array of variables that were potentially associated with moderate-to-severe depression, anxiety and PTSD-like symptoms during the pandemic. Our results showed that psychiatric patients experienced greater depressive and anxiety symptom severity but less severe PTSD-like symptoms than controls without pre-existing mental disorders. We further observed that CMD patients exhibited more severe depressive and anxiety symptoms than SMD patients and controls, whereas SMD patients displayed lower levels of PTSD-like symptoms than CMD patients and controls. These findings are generally consistent with most previous studies which found greater depressive and anxiety symptom severity in psychiatric patients relative to controls [9–14], but contrary to some other COVID-19 studies demonstrating that psychiatric patients (mixed with CMD and SMD patients in a single category) had more severe PTSD-like symptoms than those without mental disorders [11, 12, 14]. Of note, the finding of fewer PTSD-like symptoms in SMD patients than in controls in our unadjusted comparison analysis might be influenced by residual confounding, and should be treated with caution. A variety of negative social ramifications in relation to the pandemic such as life disruption, economic downturn and restricted access to psychiatric care and social support may have contributed to increased severity in depression and anxiety among psychiatric patients, particularly CMD patients, relative to people without mental disorders. Intriguingly, longitudinal research demonstrated that psychiatric patients with the greatest mental health burden displayed a significant reduction in symptom severity during the COVID-19, compared to the pre-pandemic era [42]. It is plausible that compared to the general population, individuals with high mental health burden, such as SMD patients, may be more accustomed to social isolation (e.g., limited social network and support), low functional status (e.g., sustained unemployment and poor vocational functioning) and emotional disturbance due to the inherent nature of their severe pre-existing illness [43, 44]. Hence, the pandemic-related stringent public health measures and the related adverse socio-economic impacts may appear to have narrowed the gap in psychological wellbeing and global functional levels between SMD patients and people without mental disorders.

Notably, our results revealed that more than 40% of CMD patients experienced moderate-to-severe depression, anxiety and PTSD-like symptoms, with the prevalence for depression even reaching 55%. In addition, one-fourth of SMD patients also experienced moderate-to-severe levels of depression, anxiety and PTSD-like symptoms. Our estimates are thus higher than those reported in a recent meta-analysis which indicated that 20–30% of psychiatric patients had clinically significant depressive, anxiety and PTSD-like symptoms [5]. The cumulative negative mental health impact associated with a series of recent population-level stressors in HK including social unrest in 2019 [45] and an ongoing COVID-19 pandemic, especially the fifth wave, may contribute to our observation of comparatively higher levels of psychological distress in psychiatric patients. Taken together, these findings highlight the importance of providing easily accessible psychiatric service and social support to patients with pre-existing mental disorders during COVID-19 and the future pandemics. In particular, telemedicine may provide new opportunities to address the mental health needs of psychiatric patients. In fact, earlier meta-analyses suggested that telepsychiatry was comparable to face-to-face service in terms of reliability of clinical assessment and treatment outcome [46, 47]. Recent investigations have further demonstrated positive effects of telehealth interventions on managing menta health symptoms during the pandemic [48, 49].

We sought to examine factors that were associated with moderate-to-severe depression, anxiety and PTSD-like symptoms in psychiatric patients. Our results showed that female SMD patients were significantly more likely to experience higher levels of PTSD-like symptoms than their male counterparts. This accords with a Spanish study showing that female gender was related to higher rate of avoidance, a core PTSD-like symptom, in psychiatric patients during the pandemic [9]. This finding, however, occurred only in SMD but not CMD patients. Otherwise, in line with most literature, we found lack of significant gender difference in depression and anxiety among psychiatric patients [9, 18]. We also observed that several socio-demographic characteristics, namely lower educational level, single marital status and being a housewife were independently associated with an elevated risk of depression, anxiety and/or PTSD-like symptoms in SMD patients. Regarding other mental health symptoms, our results revealed that insomnia symptoms were significantly related to depression and/or anxiety in CMD and SMD patients. More severe positive symptoms (or “psychotic-like symptoms” as measured by CAPE-C15) were found to be associated with anxiety in CMD patients. In fact, accumulating evidence has demonstrated positive relationships of psychotic-like symptoms with anxiety and depression severity, irrespective of the COVID-19 pandemic [50–52]. We further noted that CMD and SMD patients with these three adverse mental health outcomes were more likely to report greater cognitive complaints. It might be possible that the underlying cognitive impairment in psychiatric patients might be worsened by increased depressive, anxiety and/or PTSD-like symptom severity as previous research showed that cognitive dysfunction was positively correlated with depressive symptoms [53]. Our finding that self-harm behavior was associated with depression in SMD patients largely echoes with existing pandemic research indicating a significant relationship between greater depressive symptom severity and heightened risk of suicidal ideation and behavior [54].

Prior studies suggested that low resilience and the use of maladaptive coping strategies were linked to increased pandemic-related stress and psychological distress [55, 56]. Similarly, our results showed that CMD patients with greater resilience had reduced likelihood of experiencing negative mental health outcomes during the peak of the fifth COVID-19 wave in HK, whereas higher levels of engagement in avoidant coping increased the risk for moderate-to-severe anxiety among SMD patients. These findings thus underscore the importance of resilience enhancement and avoidance of adopting maladaptive coping to properly address adverse psychological impact of COVID-19. In fact, accumulating data has shown that resilience of psychiatric patients could be improved by certain psychological interventions. For instance, a recent randomized controlled trial has demonstrated that mindfulness-based cognitive therapy (MCBT) combining face-to-face group therapy sessions and self-help MBCT courses effectively enhanced psychological resilience and self-esteem in patients with schizophrenia [57]. Alternatively, we affirmed the critical role of COVID-19 related factors on influencing psychological wellbeing of psychiatric patients [18]. Our results revealed that greater burden of COVID-19 related stressors, longer exposure to pandemic-related information, and never contracting COVID-19 infection were independently associated with elevated risk for some of the negative mental health outcomes of our study.

Several methodological limitations warrant consideration in interpreting the study results. First, the cross-sectional study design precludes us from establishing causality between psychological distress and study variables. Longitudinal research is required to identify factors predicting negative mental health outcomes. Second, our patient sample was recruited from outpatient clinics only and did not include those hospitalized in psychiatric inpatient units, and may therefore introduce selection bias towards patients with milder illness severity. Third, we did not assess the use and side-effects of psychotropic medications, which may affect the likelihood of experiencing psychological distress during the pandemic. Fourth, mental health symptom assessments were based on participants’ self-reporting (albeit well-validated and commonly used in mental health surveys) which may not well align with the corresponding rating instruments administered by mental health professionals. Fifth, mental health symptom assessments were based on participants’ self-rated questionnaires, which although are commonly-used, well-validated instruments, may be subject to social desirability bias and recall errors, and may not well align with the corresponding rating instruments administered by mental health professionals. Sixth, the relatively wide 95% CIs of the estimates of the correlates associated with mental health outcomes in the final models, which may likely be due to the modest sample size, might indicate imprecise estimation, and hence the study results should be treated with caution.

In conclusion, our results showed that moderate-to-severe depression, anxiety and PTSD-like symptoms were prevalent among Chinese psychiatric patients with CMD and SMD amidst the fifth wave of COVID-19 pandemic. An array of variables encompassing socio-demographics, other mental health symptoms, psychosocial measures and COVID-19 related factors were identified as correlates of negative mental health outcomes. Provision of easily accessible psychiatric service, strengthening of resilience, encouragement of adopting adaptive coping strategies to address pandemic-related stressors, as well as early detection of psychiatric patients with the identified risk factors followed by delivery of targeted interventions would minimize the adverse mental health impact on this vulnerable population during the outbreak of infectious diseases in the future.

### Electronic supplementary material

Below is the link to the electronic supplementary material.

Supplementary Material 1

## Data Availability

The data that support the findings of this study are available from the corresponding author upon reasonable request.
